# Increasing relevance of Gram-positive cocci in urinary tract infections: a 10-year analysis of their prevalence and resistance trends

**DOI:** 10.1038/s41598-020-74834-y

**Published:** 2020-10-19

**Authors:** Márió Gajdács, Marianna Ábrók, Andrea Lázár, Katalin Burián

**Affiliations:** 1 Department of Pharmacodynamics and Biopharmacy, Faculty of Pharmacy, University of Szeged, Eötvös utca 6, Szeged, 6720 Hungary; 2 Institute of Clinical Microbiology, Faculty of Medicine, University of Szeged, Semmelweis utca 6, Szeged, 6725 Hungary; 3 Department of Medical Microbiology and Immunobiology, Faculty of Medicine, University of Szeged, Dóm tér 10, Szeged, 6720 Hungary

**Keywords:** Antimicrobials, Bacteriology, Clinical microbiology, Urogenital diseases, Urological manifestations, Epidemiology

## Abstract

Urinary tract infections (UTIs) are the third most common types of infection in human medicine worldwide. There is increasing appreciation for the pathogenic role of Gram-positive cocci (GPC) in UTIs, as they have a plethora of virulence factors, maintaining their pathogenicity and high affinity for the epithelial cells of the urinary tract*.* The study was carried out using microbiological data collected corresponding to the period between 2008 and 2017. Antimicrobial susceptibility testing was performed using the disk diffusion method and E-tests. The age range of patients affected from the outpatient and inpatient groups differed significantly (43 [range 0.7–99] vs. 68 [range 0.4–99] years; *p* = 0.008). 3962 GPCs were obtained from inpatient and 4358 from outpatient samples, corresponding to 20.5 ± 2.8% (range 17.5–26.8%) and 20.6 ± 2.6% (range 17.8–26.0%) of all positive urine samples (*p* > 0.05); in both groups, *Enterococcus* spp. were the most prevalent (outpatients: 79.6%; inpatients: 88.5%). High-level aminoglycoside resistance in enterococci was noted in 31.0–46.6% of cases. A pronounced increase in the number of MRSA was seen in the second half of the study period (0.6–1.9% vs. 9.8–11.6%; *p* = 0.038). The ratio of VRE isolates was 0.16%, no VISA/VRSA isolates were detected.

## Introduction

Urinary tract infections (UTIs; ranging from uncomplicated cystitis to severe pyelonephritis and nephrolithiasis) are the third most common types of infection in human medicine worldwide (after respiratory tract infections and infections of the alimentary tract), and the second most commonly occurring infections in developed countries, with 100–180 million cases/year^1–3^. These infections affect outpatients and hospitalized patients to a significant extent (accounting for 25–50% of hospital-acquired infections overall), representing an important factor of morbidity, especially due to their recurring nature^1,4^. UTIs more commonly affect females, patients with immunosuppression or underlying diseases/developmental abnormalities of the urinary system and they are associated with some lifestyle choices (sexual promiscuity, public baths)^1,5^. If left untreated, these infections may lead to complications, debilitating sequelae and a decreased quality of life (QoL)^6^. UTIs should also be considered an important economic undertaking, as the medical care, pharmacotherapy and lost working days corresponding to these pathologies are estimated to be around 5–7 billion US$^1,2,7^. The etiology of UTIs are thought to be predictable, due to the relatively constant spectrum of pathogens implicated, however, due to the advancements of medical interventions, pharmacotherapy and the increasing number of patients affected by immunosuppression (disease-associated or iatrogenic), other less common pathogens are now emerging as prominent factors of disease^1–6^. The most common pathogens in UTIs are the members of the Enterobacterales order (Gram-negative bacteria found in the gut, namely *Escherichia coli*, *Klebsiella* spp., pathogens of the CES group [*Citrobacter-Enterobacter-Serratia*], members of the *Proteae* tribe [*Proteus-Providencia-Morganella*]), other causative agents include Gram-positive cocci (*Enterococcus* spp., *Streptococcus* spp., *Staphylococcus saprophyticus* and *S. aureus*), non-fermenting Gram-negative bacteria (*Pseudomonas* spp. and *Acinetobacter* spp.), atypical microorganisms (*Mycoplasma, Ureaplasma* species) and yeasts (*Candida* spp.)^1–4,8–11^.

Gram-positive facultative anaerobic cocci include several phenotypically heterogenous genera from the Firmicutes phylum: *Staphycoccus* spp. are members of the Bacillales order, while *Streptococcus* (Group A, B, C and G streptococci, based on Lancefield classification) and *Enterococcus* spp. (Group D streptococci) are members of the Lactobacillales order^12^. *Staphylococcus* spp. are ubiquitously found on the skin of humans, additionally, *S. aureus* is one of the most common pathogens of severe suppurative skin and soft tissue infections, abscesses, pneumonia, endocarditis and bacteremia; methicillin-resistant *S. aureus* (MRSA) is also one of the most commonly encountered nosocomial pathogens in the US and Europe^13,14^. *S. aureus* and coagulase-negative staphylococci (CoNS) were previously considered to be uncommon etiological agents in ascending UTIs in outpatients, however, they may have a more pronounced role in hospitalized, immunocompromised patients. The isolation of *S. aureus* from urine may also be an indicator of a more severe condition (e.g., bacteremia or endocarditis), where the microorganisms reach the kidneys through hematogenous dissemination^15^. The isolation frequency of *S. aureus* from UTIs is around 0.5–13% in the literature^13,16^. In contrast, *S. saprophyticus* is a well-characterized pathogen in both uncomplicated cystitis and catheter-associated UTIs. The pathogenic role of *S. saprophyticus* in UTIs (sometimes referred to as “honeymoon cystitis”) was described in the 1960s and since then, more and more evidence was found regarding the pathogenesis of this disease^17^. Most of epidemiological studies estimate *S. saprophyticus* as causative agents in 5–20% of UTIs, however, a study from Sweden found that this pathogen was the etiological agent in > 40% of uncomplicated UTIs in females^18^. Species of the *Enterococcus* genus are abundantly found in the gut microbiota of animals and humans, being one of the few Gram-positive bacteria that are resistant to bile^19^. Enterococci are also highly prevalent in aquatic environments and should be considered as an indicator of fecal contamination in urban areas^20^. *E. faecalis* and *E. faecium* are the most common species in bacteremia, endocarditis, central nervous system infections and UTIs, however, the emergence of non-*faecalis* enterococci should be taken into consideration^21,22^. Similarly to *S. aureus*, these pathogens are relevant in nosocomial infections worldwide^23^. Temporal changes in the occurrence of Gram-positive cocci in UTIs have been described, two peaks (one in early summer, the other in the winter months) were observed in multiple studies^17,18^. The role of companion animals as reservoirs of *S. aureus* and *Enterococcus* spp. and the consideration of these pathogens as zoonotic has been published by several reports^24,25^.

The therapy of UTIs in both inpatient and outpatient settings is becoming increasingly difficult, due to the emergence of drug resistance in these pathogens, leaving clinicians with few therapeutic options available^26,27^. Gram-positive cocci are no exception to this trend: the clinical significance of methicillin-resistant *S. aureus* [MRSA] is well known, in addition, vancomycin-resistant *Enterococci* [VRE] are a significant and a sharply increasing resistance problem worldwide. One must also mention the slow, but visible emerging threat of vancomycin-intermediate *S. aureus* [VISA] species, which will be daunting challenge for therapy^25,28^. Several factors contribute to the global emergence of antimicrobial resistance (AMR), however, the overuse and misuse of antimicrobials in human and animal healthcare, in addition to globalization (allowing for fast travel to geographically distant regions of the globe, leading to the spread of multidrug resistant pathogens) may be considered as some of the most important^29^. Multiple reports have demonstrated that resistance plasmids can continuously accumulate new resistance determinants for affected bacteria without losing previous ones, therefore bacteria carrying these plasmids end up with resistance against an extensive list of available antimicrobials^30^. Nevertheless, the resulting selection pressure will fuel the “antibiotic resistance spiral”, i.e., the more pronounced use of last-resort antibiotics against drug resistant pathogens, which unavoidably leads to the emergence of resistant strains against the last resort agents (e.g., in the case of the increasing prevalence of MRSA, which lead to the use of vancomycin, corresponding to the emergence of vancomycin-intermediate *S. aureus* [VISA], VRSA and VRE isolates, leading to the use of linezolid/daptomycin)^31–33^. The increasing use of oral vancomycin in the therapy of *Clostridioides difficile* infections may put further pressure on the selection of these MDR pathogens^34^. Nevertheless, gaining more and more resistance determinants also burdens the pathogenic bacteria (i.e. they might therefore lose from their original viability and their competitiveness, see the principle of cost–benefit), thus, they may lose “ground” in their fight for the respective niche against bacteria with less resistance-determinants^35^.

The epidemiology and antibiotic-susceptibility trends of urinary tract pathogens show pronounced variation, both temporally and regionally, therefore, the assessment of these data using analytical epidemiology is essential to reflect on the national situation, compared to international data^36^. The knowledge of these resistance trends may also aid treating physicians in the optimal choice for antibiotic therapy^37^. The aim of this study was to evaluate the resistance trends and epidemiology of Gram-positive cocci in the UTIs of inpatients and outpatients at the Albert Szent-Györgyi Clinical Center (Szeged, Hungary) retrospectively, during a 10-year study period.

## Methods

### Study location and design, data collection

The present microbiological study was carried out using data collected retrospectively, regarding the time period between January 1st, 2008 and December 31st 2017, at the Institute of Clinical Microbiology, University of Szeged. The Institute is the affiliated clinical microbiology laboratory of the Albert Szent-Györgyi Clinical Center, which is a 1,820-bed primary-and tertiary-care teaching hospital in the Southern Great Plain of Hungary (population: ~ 402,000 people; 2017)^38^. Data collection was performed electronically in the records of the laboratory information system by the authors, corresponding to urine samples positive for relevant Gram-positive bacteria.

Samples with clinically significant colony counts for uropathogenic Gram-positive cocci (> 10^5^ CFU/mL; however, this was subject to interpretation by the senior clinical microbiologist, and on the basis of information provided on the clinical request forms for microbiological analysis and international guidelines) that were positive for nitrite and leukocyte-esterase tests were included in the data analysis. Only the first isolate per patient was included in the study; however, isolates with different antibiotic-susceptibility patterns (i.e. if the isolate showed different susceptibility to at least one tested antibiotic) from the same patient were considered as different individual isolates.

To evaluate the demographic characteristics of these infections, limited amount of patient data was also collected (sex, age at sample submission, date of samples submission, inpatient/outpatient status). The study was deemed exempt from ethics review by the Institutional Review Board of the University of Szeged (Szeged, Hungary), and informed consent was not required as data anonymity was maintained.

### Identification of isolates

Ten microliters of each uncentrifuged urine sample was cultured on UriSelect chromogenic agar (Bio-Rad, Berkeley, CA, USA) and blood agar (bioMérieux, Marcy-l’Étoile, France) plates with a calibrated loop, according to the manufacturer’s instructions, and incubated at 37 °C for 24–48 h, aerobically. In the period between 2008 and 2012, presumptive, biochemical reaction-based methods and VITEK 2 Compact ID/AST (bioMérieux, Marcy-l’Étoile, France) were used for bacterial identification; from 2013 onward, MALDI-TOF MS (Bruker Daltonics, Germany) was introduced to the workflow of the Department of Bacteriology. Mass spectrometry was performed by the Microflex MALDI Biotyper (Bruker Daltonics, Germany) instrument, using the MALDI Biotyper RTC 3.1 software (Bruker Daltonics, Germany) and the MALDI Biotyper Library 3.1 for spectrum analysis. Sample preparation methodology and the technical details of MALDI-TOF MS measurements were described elsewhere^39^.

### Susceptibility testing of relevant isolates

Antimicrobial susceptibility testing for the relevant Gram-positive species was performed using disk diffusion method (Liofilchem, Abruzzo, Italy) and E-tests (Liofilchem, Abruzzo, Italy) on Mueller–Hinton agar (MHA) plates, incubated at 35 ± 1 °C for 18–24 h before plate reading. The following antibiotic disks were used: penicillin (1 IU), ampicillin (2 μg for *S. saprophyticus* and 10 μg for *Enterorococcus* spp.), cefoxitin (30 μg), ceftraroline (5 μg), erythromycin (15 μg), clindamycin (2 μg), ciprofloxacin (5 μg), amikacin (30 μg), gentamicin (10 μg), nitrofurantoin (100 μg), rifampicin (5 μg), quinupristin–dalfopristin (15 μg), fusidic acid (10 μg), linezolid (10 μg), doxycycline (30 μg), tigecycline (15 μg), trimethoprim-sulfomethoxazole (23.75/1.25 μg) taking into account the intrinsic resistance mechanisms of the isolates and the clinical relevance of the listed antibiotics in the therapy of said infections^40^. The interpretation of the results was based on EUCAST breakpoints (https://www.eucast.org) valid at the time of interpretation, the re-analysis of susceptibilities based on revised breakpoints was not performed. Inducible clindamycin resistance was detected using the erythromycin-clindamycin D test, these strains were also reported as resistant to clindamycin. During data analysis, intermediately-susceptible results were grouped with and reported as resistant.

Methicillin-resistant *S. aureus* (MRSA) was detected using mannitol-salt agar (MSA) using cefoxitin disks (< 22 mm zone diameter) and PBP2′ Latex Agglutination Test Kit (Thermo Fisher Scientific Hungary Gmbh., Budapest, Hungary)^28^. After 2013, a combined MALDI-TOF MS and PBP2′ latex agglutination protocol was introduced in our laboratory^41^. MRSA-positive isolates were reported as resistant to all β-lactam antibiotics (except for 5th generation cephalosporins). Screening for high-level aminoglycoside resistance (HLAR) was done using gentamicin (30 μg) disks, while verification of positive results was performed using broth microdilution method (with a gentamicin concentration of 500 μg/ml)^42^.

*S. aureus* ATCC 29,213, *S. aureus* ATCC 43,300, *E. faecalis* ATCC 29,212, *Proteus mirabilis* ATCC 35,659, *Escherichia coli* ATCC 25,922 and *Pseudomonas aeruginosa* ATCC 27,853 were used as quality control strains^43^.

### Statistical analyses

Statistical analyses, including descriptive analysis (means or medians with ranges and percentages to characterize data) and statistical tests (χ^2^-test, Student’s t-test and Mann–Whitney U test) were performed with SPSS software version 24 (IBM SPSS Statistics for Windows 24.0, Armonk, NY, USA, IBM Corp.). The normality of variables was tested using Shapiro–Wilk tests. *p* values < 0.05 were considered statistically significant^43^.

## Results

### Demographic characteristics of affected patients, sample types

**Figure 1:**
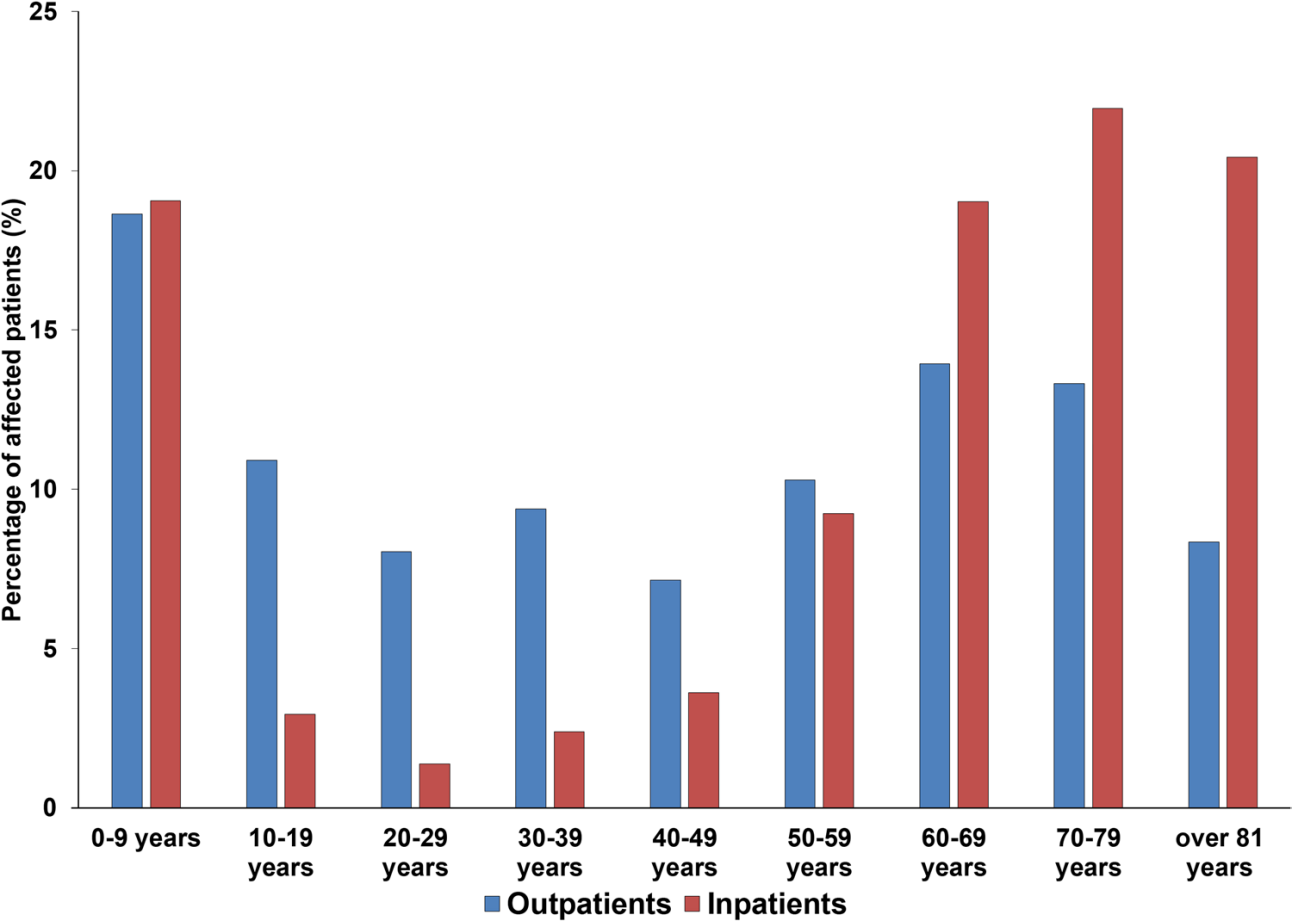
Age distribution of the affected patients in the outpatient and inpatient group.

During the study period, the median age of outpatients affected by UTIs caused by Gram-positive cocci was 43 years, which showed the following variability during the two parts of the study period: age range for outpatients was 0.7–99 years, whereas the median age for the first half of the study period was 35, while for the second half was 54 years (*p* = 0.038) (see Fig. 1. for detailed age distribution). In contrast, for the inpatients, the median age overall was 68 years; the age rage range was 0.4–99 years, with a median age for the first half of the study period was 64, while for the second half was 70 years (*p* < 0.05). The female-to-male ratio of the outpatient group was 2.03 (that is 67.1% female), and 1.15 (that is 53.6% female) in the inpatient group, respectively. The observed difference in age distribution of the two patient groups (inpatients and outpatients) was statistically significant (43 vs. 68 years; *p* = 0.008). Patients under 10 (outpatients: 18.7%, inpatients: 19.1%) and over 60 years of age (outpatients: 35.6%, inpatients: 61.4%) were predominantly affected. The sample distribution among relevant urine samples was the following: among samples from outpatient clinics, the overwhelming majority was midstream urine (98.8%), with a minority of first-stream urine (1.2%); on the other hand, the distribution from inpatient departments was more variable: midstream urine (29.2%), first-stream urine (6.3%), catheter-specimen urine (63.6%) and suprapubic bladder taps (0.9%).

### Distribution of Gram-positive cocci in urine samples

**Figure 2:**
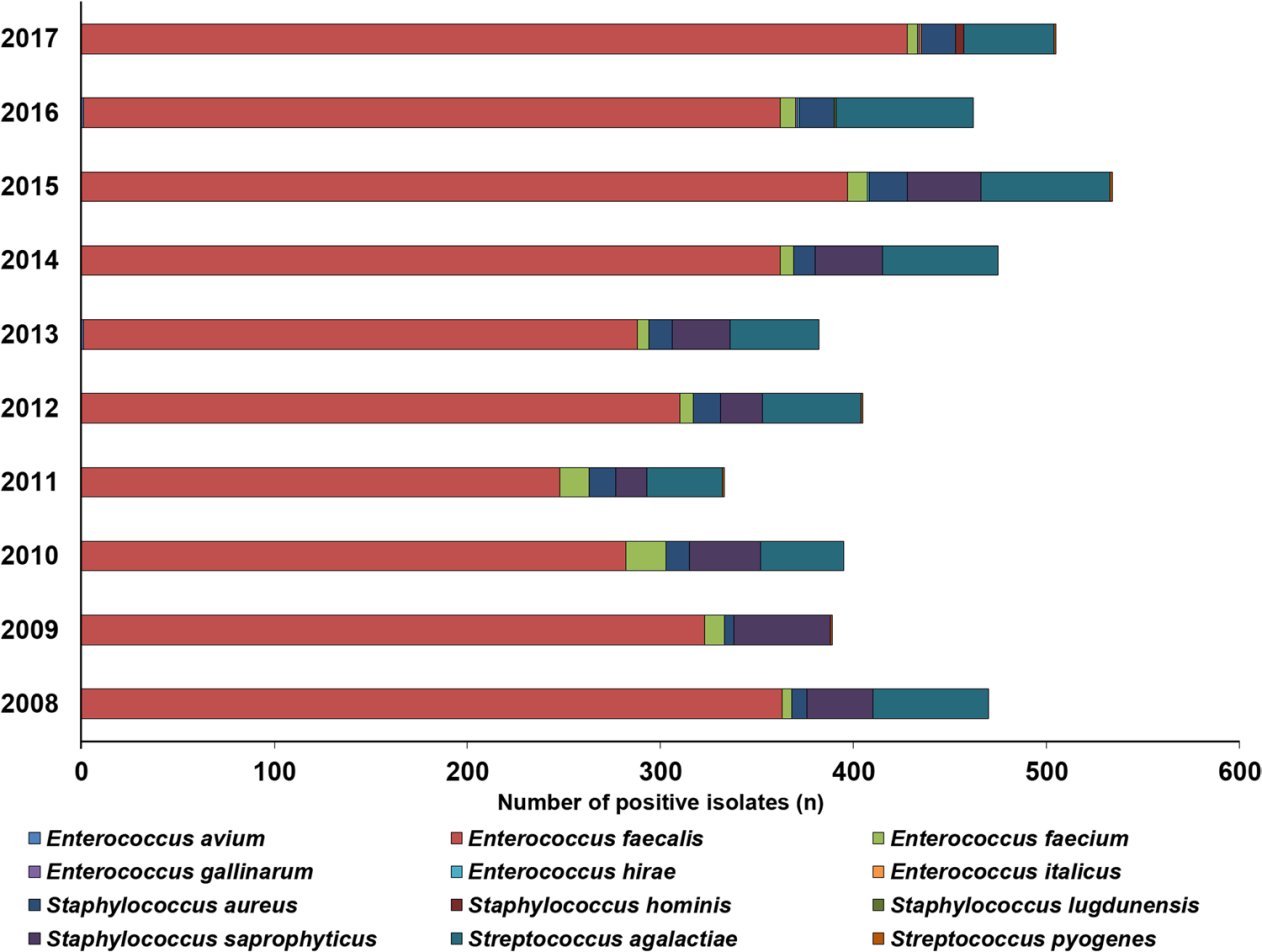
Frequency and species distribution of Gram-positive bacterial isolates in outpatient samples (2008–2017).

**Figure 3:**
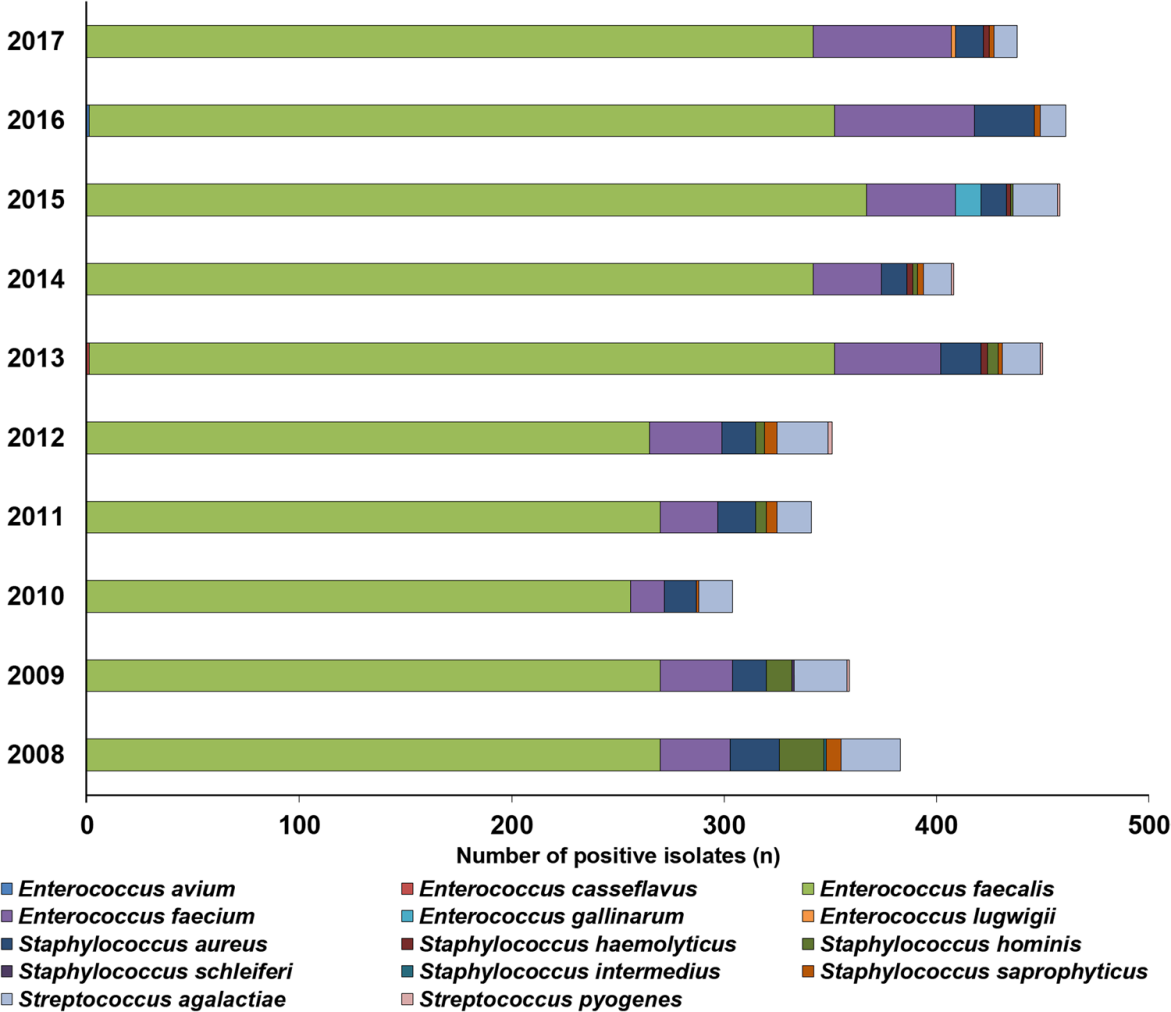
Frequency and species distribution of Gram-positive bacterial isolates in inpatient samples (2008–2017).

Between 2008 and 2017, the Institute of Clinical Microbiology received 21,150 urine samples from outpatient clinics and 19,325 samples from inpatient departments, from which a significant urinary pathogen was detected. 3962 Gram-positive coccus isolates were obtained from inpatients (396.2 ± 54.2/year) and 4358 from outpatients (435.8 ± 64.6/year). This corresponds to 20.5 ± 2.6% (range 17.8–26.0%) for outpatients, while 20.6 ± 2.8% (range 17.5–26.8%) of all positive urine samples for inpatients; (*p* > 0.05). In both groups, *Enterococcus* spp. (predominantly *E. faecalis*; outpatients: 79.6%; inpatients: 88.5%) were the most prevalent, while *Staphylococcus* spp. (outpatients: 9.2%, mainly *S. saprophyticus*; inpatients: 6.7%, mainly *S. aureus*) and *Streptococcus* spp. (predominantly *S. agalactiae*; outpatients: 11.2%; inpatients: 4.8%) were in a minority. The epidemiology and detailed species distribution of Gram-positive cocci in both patient groups are presented in Fig. 2 (outpatients) and Fig. 3 (inpatients). There was an obvious seasonal trend in the isolation of Gram-positive cocci in the outpatient group (24.7% was isolated in the June–July periods, 22.9% in the December-January periods), while no such tendency was noted in the inpatient group. In the inpatient group, 14 different species of Gram-positive urinary pathogens were isolated (median 7; range 5–9), while in the outpatient group, the species distribution was less diverse, with 12 different species (median 6; range 5–8) detected.

### Antibiotic resistance trends among Gram-positive cocci isolated from UTIs

Antibiotic resistance data of the isolated enterococci, staphylococci and streptococci in the 10-year study period is presented in Tables 1, 2 and 3, respectively. To identify temporal developments in resistance trends, the 10-year study period was divided into two 5-year periods (2008–2012 and 2013–2017). The level of resistance in *Enterococcus* species was significantly higher in isolates originating from inpatients in both periods regarding ciprofloxacin, but not other antibiotics. Apart from intrinsic resistance, resistance rates against ciprofloxacin and HLAR did not show relevant differences among *E. faecalis* and non-faecalis enterococci (*p* > 0.05). Likewise, there was no significant increase noted in the ratio of resistance strains in either patient groups between the two 5-year periods. HLAR was detected in 31.0–46.6% of isolates overall and there was a numerical, but not significant increase in the second half of the study period (*p* = 0.067). Very few VRE isolates were recovered (0.16%; n = 11 from inpatients and n = 4 from outpatients, exclusively from *E. faecium*), while no linezolid-resistant isolates were detected (Table 1.).

**Table 1:** Percentage of resistant *Enterococcus* strains to indicator antibiotics from inpatient and outpatient departments (2008–2017).

	2008–2012			2013–2017		
	Outpatients (%)	Inpatients (%)	Statistics^a^	Outpatients (%)	Inpatients	Statistics^a^
Ampicillin^b^	0.1	0.3	n.s	0.2	0.4%	n.s
Imipenem^b^	0.2	0.2	n.s	0.2	0.2%	n.s
Ciprofloxacin	31.6	45.2	*p* = 0.026	16.1%	33.0	*p* = 0.019
HLAR^c^	31.0	39.8	n.s	45.8%	46.6	n.s
Vancomycin^d^	0.0	0.1	n.s	0.1%	0.3	n.s
QP/DP^e^	0.0	0.0	n.s	0.0%	0.0	n.s
Tigecycline	0.0	0.0	n.s	0.0%	0.0	n.s
Linezolid	0.0	0.0	n.s	0.0%	0.0	n.s

^a^Comparison of resistance levels among isolates originating from outpatients and inpatients.

^b^Calculated for *E. faecalis* isolates only.

^c^High-level aminoglycoside resistance.

^d^Represents the ratio of VRE strains.

^e^Quinpristin/dalfopristin.

*n.s.* not significant (*p* > 0.05).

**Table 2:** Percentage of resistant *Staphylococcus* strains to indicator antibiotics from inpatient and outpatient departments (2008–2017).

	2008–2012			2013–2017		
	Outpatients (%)	Inpatients (%)	Statistics^a^	Outpatients (%)	Inpatients (%)	Statistics^a^
Penicillin	94.8	96.6	n.s	95.2	96.9	n.s
Ampicillin^b^	10.8	12.2	n.s	12.4	13.7	n.s
Cefoxitin^c^	0.6	1.9	n.s	9.8	11.6	n.s
Amikacin	1.5	23.2	*p* < 0.001	1.1	14.2	*p* < 0.001
Gentamicin	4.6	23.2	*p* = 0.028	2.1	24.8	*p* < 0.001
Azithromycin	19.5	25.8	*p* = 0.049	32.3	33.2	n.s
Clindamycin	17.9	21.9	n.s	22.1	26.3	n.s
Ciprofloxacin	9.7	35.7	*p* < 0.001	12.1	25.6	*p* = 0.042
Doxycycline	13.3	29.8	*p* = 0.04	4.2	30.8	*p* < 0.001
Nitrofurantoin	2.6	3.3	n.s	0.5	3.8	*p* = 0.046
SMX/TMP^d^	5.6	23.2	*p* < 0.001	1.1	27.1	*p* < 0.001
Rifampicin	1.0	2.3	n.s	3.3	4.8	n.s
Vancomycin^e^	0.0	0.0	n.s	0.0	0.0	n.s
QP/DP^f^	0.0	0.0	n.s	0.0	0.0	n.s
Tigecycline	0.0	0.0	n.s	0.0	0.0	n.s
Linezolid	0.0	0.0	n.s	0.0	0.0	n.s
Fusidic acid	0.0	0.0	n.s	0.0	0.0	n.s

^a^Comparison of resistance levels among isolates originating from outpatients and inpatients.

^b^Calculated for *S. saprophyticus* isolates only.

^c^Represent the ratio of MRSA isolates in *S. aureus.*

^d^Sulfamethoxazole-trimethoprim.

^e^Represents the ratio of VISA/VRSA strains.

^f^Quinpristin/dalfopristin.

n.s.: not significant (p > 0.05).

**Table 3:** Percentage of resistant *Streptococcus* strains to indicator antibiotics from inpatient and outpatient departments (2008–2017).

	2008–2012			2013–2017		
	Outpatients (%)	Inpatients (%)	Statistics^a^	Outpatients (%)	Inpatients (%)	Statistics^a^
Ampicillin	0.0	0.0	n.s	0.0	0.0	n.s
Azithromycin	16.0	17.8	n.s	19.4	21.3	n.s
Clindamycin	14.2	18.5	n.s	19.1	20.4	n.s
Ciprofloxacin	7.1	25.4	*p* < 0.001	8.3	29.9	*p* = 0.038
SMX/TMP^b^	3.3	19.4	*p* = 0.031	2.6	20.8	*p* < 0.001
Rifampicin	0.8	1.3	n.s	0.6	0.9	n.s
Vancomycin	0.0	0.0	n.s	0.0	0.0	n.s
Linezolid	0.0	0.0	n.s	0.0	0.0	n.s

^a^Comparison of resistance levels among isolates originating from outpatients and inpatients.

^b^Sulfamethoxazole-trimethoprim.

*n.s.* not significant (*p* > 0.05).

The resistance levels in inpatient *Staphylococcus* samples were significantly higher for amikacin, gentamicin, azithromycin (in the 2008–2012 period), ciprofloxacin, doxycycline, nitrofurantoin (in the 2013–2017 period) and trimethoprim-sulfamethoxazole (SMX/TMP). There was no significant increase in the resistance of any tested antibiotics between 2008–2012 and 2013–2017, however, a numerical tendency was found for azithromycin (*p* = 0.071). The difference in the number of MRSA isolates among inpatient and outpatients was not significant, however, a pronounced increase in the number of MRSA was seen in the second half of the study period (0.6–1.9% vs. 9.8–11.6%; *p* = 0.038). No VISA/VRSA strains were found, in addition, none of the *Staphylococcus* strains were resistant to the supplementary antibiotics (quinpristin/dalfopristin, tigecycline, linezolid, fusidic acid) (Table 2.). In the case of streptococci, significant differences were observed in the resistance levels of ciprofloxacin and SMX/TMP, but not in the case of other antibiotics. Additionally, no significant temporal changes were noted between the two study periods. No vancomycin or linezolid-resistant strains were detected.

## Discussion

The study presents the epidemiological trends and resistance levels of Gram-positive cocci in urinary tract infections (UTIs) in the southern part of Hungary, over a long surveillance period (10 years). Previous local studies have highlighted the epidemiological situation of other pathogens locally: *E. coli* (56.7% in outpatients, 42.0% in inpatients) and *Klebsiella* spp^43^. (8.9% vs. 13.0%) were detected in highest numbers, while members of the CES group^44^ (2.6% vs. 3.0%), *Proteae*^45^ (5.0% vs. 7.2%), non-fermenting Gram-negatives^46^ (3.4% vs. 5.5%) and *Candida* spp^47^. (0.4% vs. 6.0%) were present in lesser numbers. In contrast, these pathogens represented ~ 20% of the etiological agents in UTIs, both for inpatients and outpatients, therefore, their epidemiological significance should not be disregarded. Among the group of Gram-positive cocci, *E. faecalis* was the predominant species (~ 80% of isolates), which is not surprising, in light of global epidemiological reports on the causative agents for UTIs^36^. To the best of our knowledge, this is the longest-spanning study reporting on the prevalence and susceptibility of this group of uropathogens in Hungary. In contrast to previous studies, dating back some 20–30 years (where the reported prevalence of *Enterococcus* spp., *S. aureus* and *S. saprophyticus* was 2–20%, 0.2–6% and 0.5–8%, respectively), based on current literature results, their prevalence is around 8–35%, 0.5–13% and 5–20%, respectively^48–55^. This increase is prevalence is especially notable in patients affected by recurrent UTIs (recurrence 3 or more times in 6 months)^44^. The increased prevalence of these pathogens may be attributed to the increase in patients with lifestyle diseases (kidney diseases, diabetes), immunosuppression, patients undergoing surgical interventions^56^. Additionally, some reports suggest that *Enterococcus* spp. may be considered an indicator of more severe pathologies (e.g., diabetes, abnormality of the genitourinary tract)^18–22,49,56^. An overview of the literature published in the last 20 or so years, regarding the prevalence of UTIs caused by Gram-positive cocci outside Hungary is presented in Table 4. In contrast to the present report, most of these studies found that the prevalence of Gram-positive cocci was higher in inpatients (hospital-acquired infections). Of note, the seasonal occurrence/accumulation of these bacteria (particularly *S. saprophyticus*) was also verified by our results in the Southern Great Plain of Hungary.

**Table 4:** Literature summary on the prevalence of Gram-positive cocci in urinary tract infections outside Hungary (1997–2019).

First author	Study year	Country	Prevalence of Gram-positive cocci (%), most common isolate	Comments
Barros et al.^56^	1997–2005	Brazil	6.2%; *E. faecalis*	
Kothari et al.^57^	2005	India	9.6%; *S. saprophyticus*	CA-UTIs only
Toner et al.^58^	2005–2014	United Kingdom	14.7%; *E. faecalis*	9.8% of isolates were resistant to vancomycin
Behzadi et al.^11^	2007	Iran	January-March 2007: 9.1%; *Enterococcus* spp. October-December 2007: 10.6%; *Enterococcus* spp.	
Parameswarappa et al.^59^	2007–2009	India	12.1%; *Enterococcus* spp.	
Sorlózano-Puerto et al.^60^	2011–2014	Spain	22.4%; *E. faecalis*	Children only
Zarb et al.^49^	2010	European Union	17.2%; *E. faecalis*	HA-UTIs only
Lewis et al.^50^	2013	South Africa	10.8%; *E. faecalis*	CA-UTIs only
Baral et al.^51^	2013	Nepal	21.7%; *S. aurues*	
Goel et al.^61^	2013–2014	India	0.5%; *Enterococcus* spp.	CA-UTIs only
Prashamsa et al.^52^	2015	India	12.5; *E. faecalis*	
Dougnon et al.^54^	2016	West Africa	21.0%; *E. faecalis*	
Bardoroi et al.^55^	2017	India	26.7%; *S. aureus*	HA-UTIs only
Zaha et al.^62^	2017–2018	Romania	7.3%; *Enterococcus* spp. 4.9%; *Staphylococcus* spp.	All patients were affected by diabetes
Petca et al.^63^	2018	Romania	18.7%; *Enterococcus* spp.	
Urmi et al.^64^	2018	Bangladesh	8.2%; Gram-positive cocci	
Shrestha et al.^4^	2018	Nepal	21.3%; *E. faecalis*	
Petca et al.^65^	2018–2019	Romania	13.3%; *Enterococcus* spp. 2.1%; *Staphylococcus* spp.	Three different centers

*CA-UTI* community-acquired urinary tract infection, *HA-UTI* hospital-acquired/catheter-associated urinary tract infection.

Although the susceptibility-reporting for some of the antibiotics (e.g., fusidic acid, rifampicin, erythromycin, clindamycin, doxycycline and tigecycline) might seem frivolous in the context of the therapy of UTIs (as these drugs are not used in the therapy of these infections), the reporting of these results for epidemiological purposes is of interest, especially because not many studies are available regarding GPCs as uropathogens from Europe^66^. The prevalence of MRSA/VRSA and VRE isolates from urinary samples was advantageous in our study, and the levels of these isolates were similarly low in other literature reports as well, especially if we compare resistance levels of urinary isolates with invasive isolates (vancomycin-resistant *E. faecalis*: 0.0% [2008], 0.4% [2017]; vancomycin-resistant *E. faecium*: 2.8% [2008], 28.3% [2017]; MRSA: 22.5% [2008], 23.6% [2017], data from the European Antimicrobial Resistance Surveillance Network [EARS-Net])^48–56,67,68^. On the other hand, the resistance levels to auxillary antimicrobials (aminoglycosides, fluoroquinolones) was shown to be high, and presenting in an increasing tendency (HLAR in enterococci: 53.3% [2008], 62.0% [2017], data from EARS-Net)^48–56,67,68^. *S. saprophyticus* is a common agent in UTIs, however, regarding its resistance patterns, it has proven so far to be mostly sensitive to the relevant antibiotics. Among the tested antibiotics, the highest levels of resistance were detected for ciprofloxacin and SMX/TMP, which could a consequence of their prevalent use, due to their broad-spectrum activity against both Gram-positive and Gram-negative bacteria. Out of the agents effective against Gram-positive bacteria, azithromycin and clindamycin had the highest resistance levels. Enterococci are a therapeutic challenge in general, because of their intrinsic resistance mechanisms against many antibiotics (aminoglycosides, cephalosporins), and due to their genetic plasticity, they can easily acquire additional resistance determinants against other antimicrobial drugs. This is especially concerning, as these bacteria normally live in the gastrointestinal tract, where they can pick up resistance plasmids from other members of the commensal flora^69–72^. Vancomycin resistance in *Enterococcus* species is therefore a severe therapeutic issue^19–23,25^. High-level aminoglycoside-resistance (HLAR) in *Enterococcus* spp. was detected in > 40% in the second part of the study period. This resistance is usually mediated by aminoglycoside-modifying enzymes (e.g., acetyltransferases, phospho-transferases and nucleotidyl-transferases). The detection of HLAR is relevant in antimicrobial therapy, for the combined use of a cell wall-acting agent (ampicillin, imipenem) and the aminoglycoside for their pharmacological synergy^71,72^.

Gram-positive cocci have a plethora of virulence determinants, maintaining their high affinity for the epithelial cells of the urinary tract, allowing for their survival. These virulence factors include fibrillar proteins (Ssp) mediating cell–cell interactions, fibronectin-binding proteins, elastin-binding protein, adhesins, hemagglutinin, elastase and lipase. In addition, most of *S. saprophyticus* and > 90% of *S. aureus* strains produce urease, breaking down carbamide (urea) in the urine^11–23^. Staphylococci may colonize the rectum, while *Enterococcus* spp. are present in fecal matter, therefore their anatomical proximity to the urinary tract may additionally enhance their UTI-causing capabilities^20,28^. Biofilm-production in these species is an another important factor for the emergence and the persistence of UTIs, with some reports suggesting that some 80% of uropathogenic Gram-positive cocci are biofilm-producers^48^. The presence of biofilm in urethral stents and catheters may lead to obstruction; furthermore, microorganisms embedded in biofilm may survive 1000-times higher concentrations of antibiotics, compared to non-embedded (i.e. planktonic) cells^48,73,74^. The diversification and time-dependent use of these virulence determinants allows for the infectivity and survival of these bacteria. At the onset of infection (i.e. low population density), the expression levels of adhesins is more significant, while if high population densities are achieved, genes corresponding for toxin secretion are activated^74^.

Several limitations of this present study need to be acknowledged. In addition to the retrospective study design, the authors were unable to access the charts of the individual patients affected, therefore the correlation between the existence of clinically relevant risk factors (apart from age, inpatient/outpatient status, and catheterization) could not be assessed. The clear differentiation between Gram-positive asymptomatic bacteriuria and clinically significant (symptomatic) urinary tract infection in the elderly is very difficult. Furthermore, the molecular characterization and genotyping of the isolates species (which could have provided us with important data, especially in case of MRSA or *S. saprophyticus* isolates) was not performed, due to financial constraints. Also, the selection bias of publication should also be noted, as most studies describing the prevalence of infectious diseases are tertiary-care centers or specialized centers, corresponds to patients with more severe conditions or underlying illnesses^75^. Nevertheless, the information presented in this report should be useful in both national and international comparisons for epidemiological purposes; additionally, the resistance trends presented here may aid clinicians in the selection of appropriate antimicrobial therapy^76^.

## Conclusions

Although urinary tract infections are principally caused by Gram-negative bacteria, Gram-positives have emerged as important causative agents of UTIs, particularly among elderly patients, often associated with co-morbidities, pregnant women and catheterized patients, both in low- and high-income countries. In our study, corresponding to the southern region of Hungary, their prevalence was found to be around 20%, with *Enterococcus* spp. in highest numbers. While there was no relevant difference is their prevalence among inpatients and outpatients, the emergence of drug resistance in these pathogens to commonly used antibiotics is a worrisome finding, compromising therapeutic options in the clinical practice and leading to the use of agents with less advantageous side effect profiles, further contributing to the selection pressure on these microorganisms. In our local settings, the resistance rates for fluoroquinolones are particularly concerning (these agents are not recommended to be used empirically), in addition, the same goes for the use of most aminoglycosides for hospitalized patients. In contrast, the use of nitrofurantoin for staphylococci may still be regarded as safe in our settings, and the tested isolates are almost uniformly susceptible to the available last-resort antibiotics.
